# Supplementary material for: Massively Parallel Screening of Toll/Interleukin‐1 Receptor (TIR)‐Derived Peptides Reveals Multiple Toll‐Like Receptors (TLRs)‐Targeting Immunomodulatory Peptides
Source: Adv Sci (Weinh). 2024 Oct 31;12(1):2406018. doi: 10.1002/advs.202406018 (PMC11714206; doi:10.1002/advs.202406018)
Supplement: Supplementary file 1 — Supporting Information [file ADVS-12-2406018-s001.docx]

Supplementary Materials for

**Massively Parallel Screening of Toll/interleukin-1 receptor (TIR)-derived Peptides Reveals Multiple Toll-like receptors (TLRs)-targeting Immunomodulatory Peptides**

Yun Lim, Tae Kyeom Kang, Meong Il Kim, Doyhyeon Kim, Ji Yul Kim, Sang Hoon Jung, Keunwan Park, Wook-Bin Lee, & Moon-Hyeong Seo

Correspondence to: mhseo@kist.re.kr, wblee@kist.re.kr, keunwan@kist.re.kr

**This PDF file includes:**

Figures S1 to S3

Tables S1 to S6

Captions for Data S1 to S4

**Other Supplementary Materials for this manuscript include the following:**

Data S1 to S4


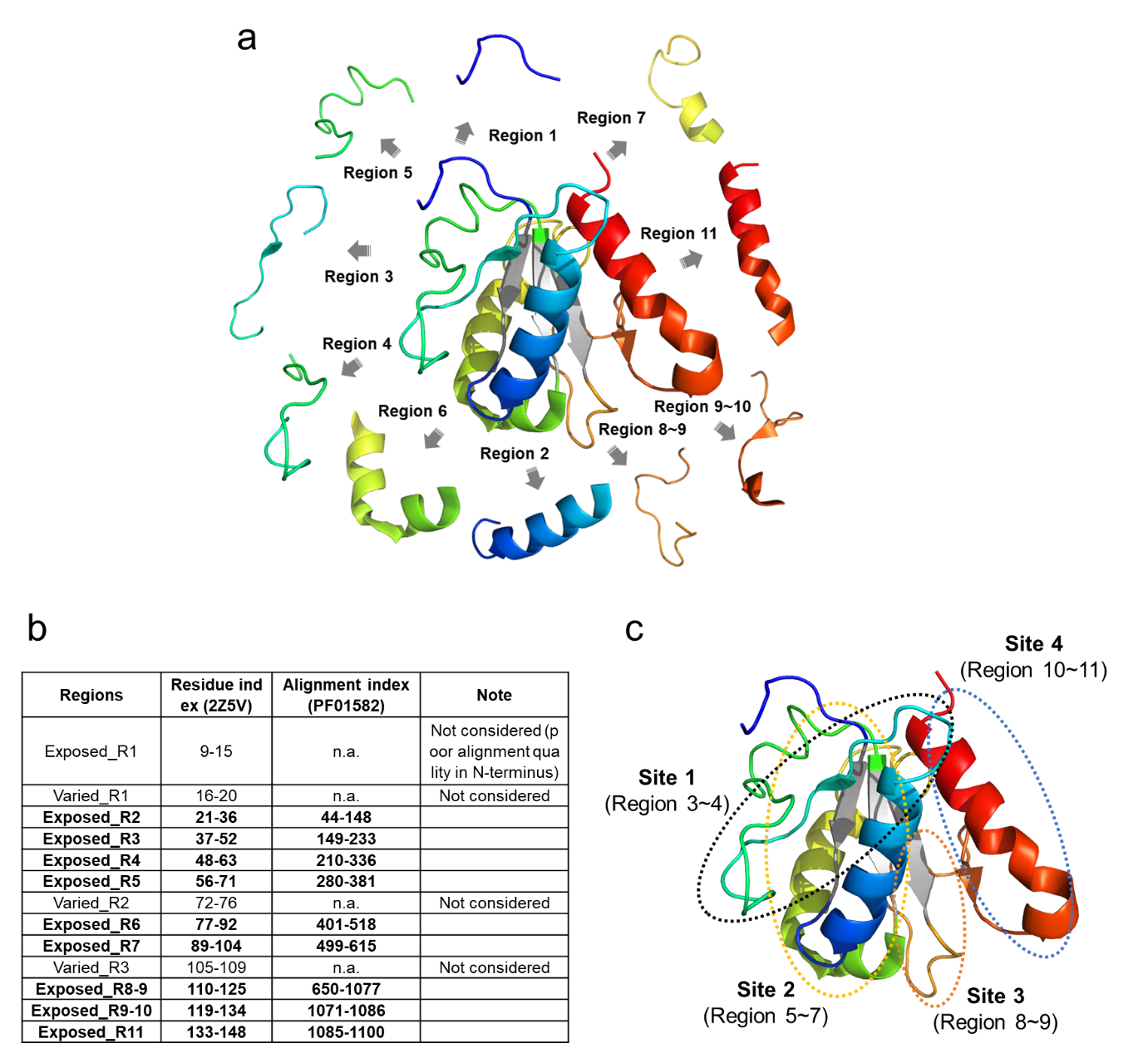


Figure. S1. Structural elements of MyD88 TIR domain. (a) Surface-exposed regions 1 to 11 of MyD88 (PDB ID 2Z5V) are shown in different colors.^[1]^ The hydrophobic core formed by parallel β-sheets (βA, βC, and βD), which is excluded in the library design, is colored in gray. (b) The extracted regions of TIRs are summarized. (c) Four sites of TIR interfaces, which mediate the interactions between TIR domains, are marked.


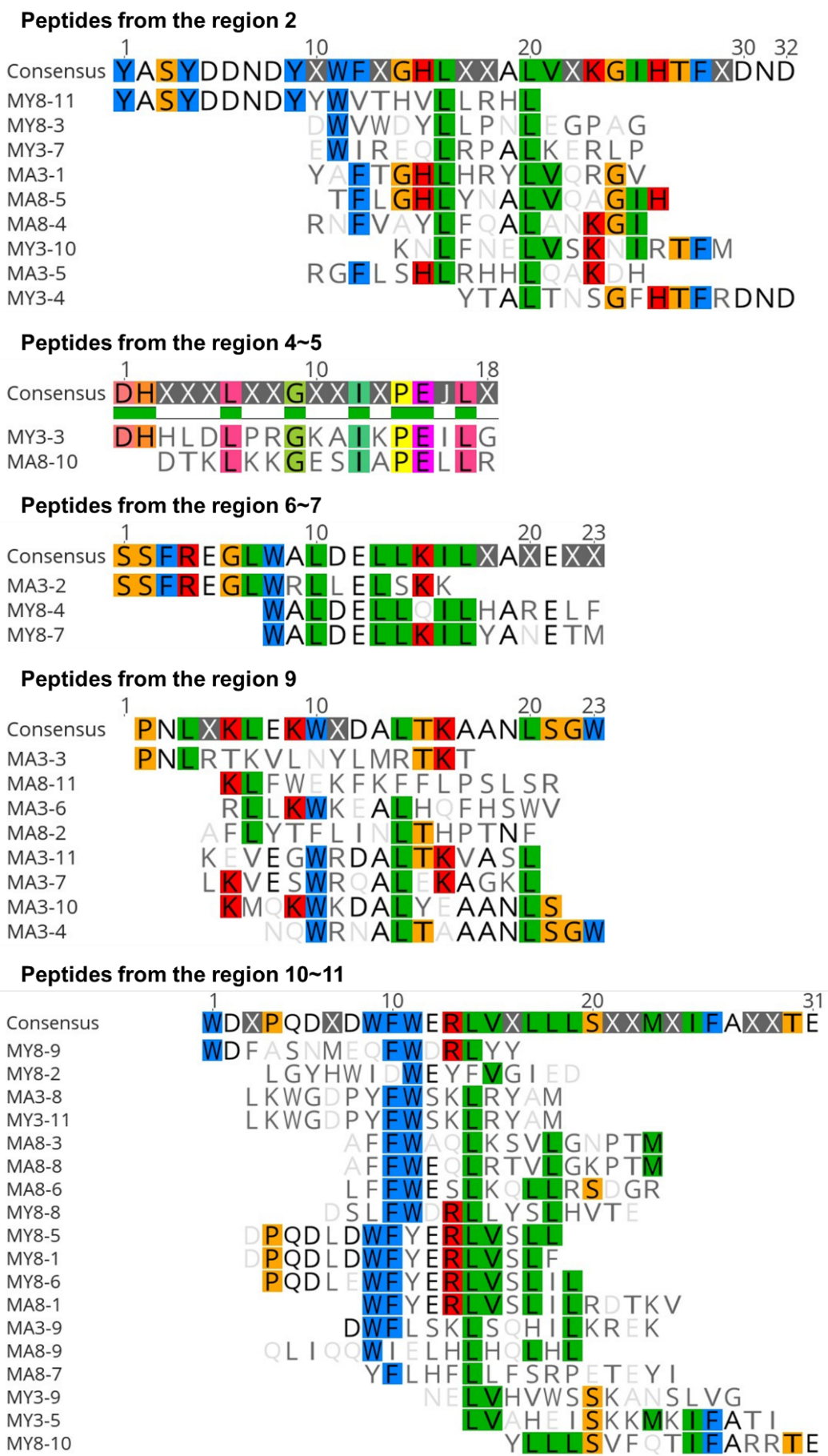


**Figure. S2.** **Sequence alignment of the selected TDIPs.** The selected peptides were aligned according to the structural regions from which the peptides originated.


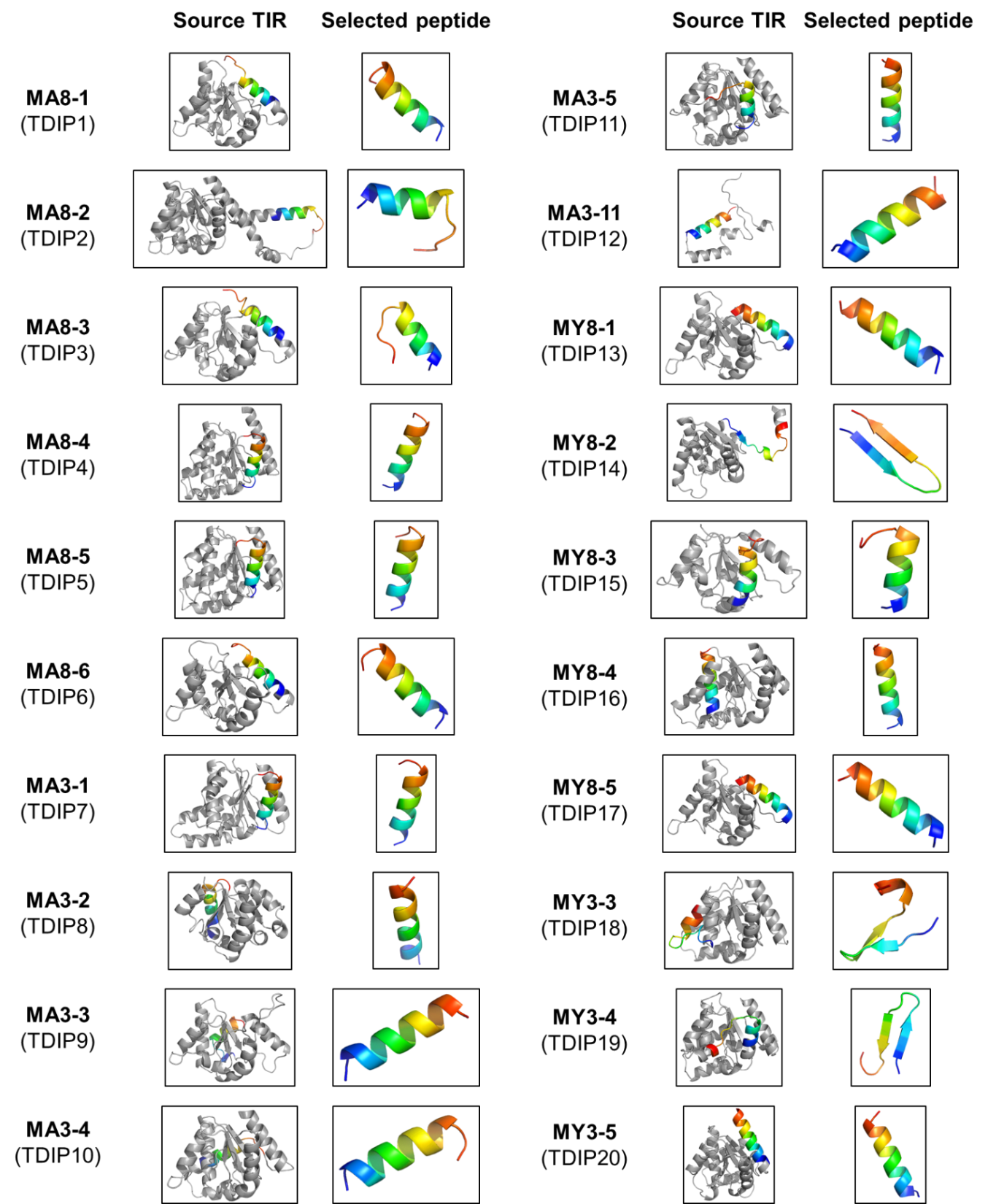


**Figure. S3.** **Structural description of the selected synthetic peptides.** The selected peptides are rainbow-colored in each AlphaFold-predicted or Swiss model structure of the source TIR domains from UniProt DB. The three-dimensional structures of the selected peptides were predicted by using PEP-FOLD4.

**Table S1. Hand-curated domains included in the T-Surf library.**

| **Uniprot ID** | **Uniprot Entry Name** | **Protein name** | **Gene Names** | **Organism** |
| --- | --- | --- | --- | --- |
| P14778 | IL1R1_HUMAN | Interleukin-1 receptor type 1 | IL1R1 | Homo sapiens (Human) |
| Q15399 | TLR1_HUMAN | Toll-like receptor 1 | TLR1 | Homo sapiens (Human) |
| O60603 | TLR2_HUMAN | Toll-like receptor 2 | TLR2 | Homo sapiens (Human) |
| O15455 | TLR3_HUMAN | Toll-like receptor 3 | TLR3 | Homo sapiens (Human) |
| O00206 | TLR4_HUMAN | Toll-like receptor 4 | TLR4 | Homo sapiens (Human) |
| O60602 | TLR5_HUMAN | Toll-like receptor 5 | TLR5 | Homo sapiens (Human) |
| Q9Y2C9 | TLR6_HUMAN | Toll-like receptor 6 | TLR6 | Homo sapiens (Human) |
| Q9NYK1 | TLR7_HUMAN | Toll-like receptor 7 | TLR7 | Homo sapiens (Human) |
| Q9NR97 | TLR8_HUMAN | Toll-like receptor 8 | TLR8 | Homo sapiens (Human) |
| Q9NR96 | TLR9_HUMAN | Toll-like receptor 9 | TLR9 | Homo sapiens (Human) |
| Q99836 | MYD88_HUMAN | Myeloid differentiation primary response protein MyD88 | MYD88 | Homo sapiens (Human) |
| P58753 | TIRAP_HUMAN | Toll/interleukin-1 receptor domain-containing adapter protein | TIRAP MAL | Homo sapiens (Human) |
| Q8IUC6 | TCAM1_HUMAN | TIR domain-containing adapter molecule 1 | TICAM1 (TRIF) | Homo sapiens (Human) |
| Q86XR7 | TCAM2_HUMAN | TIR domain-containing adapter molecule 2 | TICAM2 (TRAM) | Homo sapiens (Human) |
| Q9Y4K3 | TRAF6_HUMAN | TNF receptor-associated factor 6 | TRAF6 | Homo sapiens (Human) |
| A0A0H2V8B5 | TCPC_ECOL6 | NAD(+) hydrolase TcpC | TcpC | Escherichia coli O6:H1 (strain CFT073 / ATCC 700928 / UPEC) |
| C0RGW8 | BTPA_BRUMB | Probable 2' cyclic ADP-D-ribose synthase TcpB | TcpB | Brucella melitensis biotype 2 (strain ATCC 23457) |
| Q01220 | A52_VACCW | Protein A52 | A52R | Vaccinia virus (strain Western Reserve) (VACV) |
| P26672 | A46_VACCW | Protein OPG176 | A46R | Vaccinia virus (strain Western Reserve) (VACV) |

**Table S2. Summary of the designed T-Surf library.**

| **Designed T-Surf library** | |
| --- | --- |
| Total number of TIR domain family (Pfam PF01582) | 13,644 |
| Number of domains containing at least one peptide in the designed library | 13,603 (99.7%) |
| Total number of amino acids of TIR domain family (Pfam PF01582) | 2,164,918 |
| Number of amino acids that the T-Surf library covers | 1,529,164 (70.6%) |

**Table S3. Statistics of the T-Surf library.** The number of designed peptides in the P8 and P3 libraries are summarized. The coverage and the total diversity of the libraries are given at the peptide level.

| **NGS analysis** | | | | | |
| --- | --- | --- | --- | --- | --- |
| **Library** | **Designed peptides** | **Validated peptides** | **Coverage** | **Peptide variants ^a^** | **Total diversity** |
| **T-Surf P8** | 190,945 | 190,419 | 99.7% | 997,373 | 1,187,792 |
| **T-Surf P3** | 190,945 | 104,572 | 54.8% | 603,588 | 708,160 |

a) Non-designed peptides with mutations that were incorporated during the oligo synthesis or cloning steps.

**Table S4. The redundant peptides in the T-Surf P8 library.**

| **T-Surf P8** (Total NGS count: 25,464,130) | | | |
| --- | --- | --- | --- |
| **Peptide sequence** | **Peptide ID** | **NGS count** | **Population** |
| ANAIKHANITTFFDDD | A0A2J6JQE8_LACSA/23-207_[21-36] | 6115 | 0.0240% |
| KQKHGSIRWKEDSAEK | A0A402E7G2_9SAUR/761-938_[122-137] | 6100 | 0.0240% |
| ELVKIMEAKEKEIGHI | A0A3Q7EFL2_SOLLC/18-194_A0A3Q7EFL2.1_[7] | 5945 | 0.0233% |
| RNGFAGHLYKALARRK | V7C5I8_PHAVU/11-182_[12-27] | 5861 | 0.0230% |
| SRKYLASKWRDFELNM | K1QVT6_CRAGI/4-147_K1QVT6.1_[6] | 5835 | 0.0229% |
| DVFVSFRGEDIRRRLL | A0A151SCE5_CAJCA/10-135_[2-17] | 5723 | 0.0225% |
| DVFLNFRGIDLRSGFL | A0A371HPB5_MUCPR/11-183_[2-17] | 5671 | 0.0223% |
| SEFYDVDPSEVIERKR | A0A3N7G1G5_POPTR/21-210_[100-115] | 5668 | 0.0223% |
| NGNTSYHLALHYRDIP | A0A194RA63_PAPMA/1026-1131_A0A194RA63.1_[3] | 5526 | 0.0217% |
| QVQNRWSRALSHVSNI | D7KZ23_ARALL/16-193_[122-137] | 5300 | 0.0208% |
| RGIATFQDGQLSRGIA | A0A2P6PS83_ROSCH/23-202_[24-39] | 5262 | 0.0207% |
| LAELKSSYLPVTNYLV | A0A059CQT6_EUCGR/93-197_[87-102] | 5249 | 0.0206% |
| GEIEGGHGQRYTGRFS | A0A2T7NI39_POMCA/704-863_[26-41] | 5168 | 0.0203% |
| EELMPEANRNVYGDND | W4XXW4_STRPU/733-878_[20-35] | 5086 | 0.0200% |
| ANFTGMVFKDGYESKF | A0A2J6LLJ3_LACSA/14-203_[148-163] | 5047 | 0.0198% |
| NELAMLAKLKSSLDRR | D7KPJ9_ARALL/175-350_D7KPJ9.1_[7] | 5037 | 0.0198% |
| HFKLAVHFRDFLAGIP | R7UWB2_CAPTE/8-141_[28-43] | 4764 | 0.0187% |
| VQKWRDALKDIANLSG | A0A4P1RJZ4_LUPAN/14-193_[127-142] | 4733 | 0.0186% |
| LWRDSLREVANLSGLD | A0A0R0J0J4_SOYBN/16-148_[114-129] | 4571 | 0.0180% |
| GPTDNLDPELKTYLSM | A0A195CSW9_9HYME/844-999_[100-115] | 4568 | 0.0179% |
| LQDGYESQFIQTIVKE | B9RVC7_RICCO/19-194_[145-160] | 4528 | 0.0178% |
| DELVKMNKLADLGKLR | R0H4S0_9BRAS/197-347_R0H4S0.1_[7] | 4389 | 0.0172% |
| SFRGNDVRDGFLGKLY | G7JLU8_MEDTR/9-196_[6-21] | 4381 | 0.0172% |
| GWELKNTANGGILSKL | A0A2J6JYH2_LACSA/14-208_[137-152] | 4363 | 0.0171% |
| RDEPKISKGKSISGEL | A0A251N8H2_PRUPE/18-195_[33-48] | 4301 | 0.0169% |
| DRELPNGEEISPRLYK | A0A2U1MQH6_ARTAN/11-134_A0A2U1MQH6.1_[4] | 4239 | 0.0166% |
| EHFRALGMIEKVQRWR | A0A1U8ALK0_NELNU/16-194_[118-133] | 4231 | 0.0166% |
| ALASYAGWDVRNKPEF | A0A371EM70_MUCPR/47-226_[136-151] | 4222 | 0.0166% |
| EEVSKKINRSPLHVAN | A0A1S3UVH7_VIGRR/18-195_[161-176] | 4211 | 0.0165% |
| GTDINPKLLRAIDQSM | A0A498JXL3_MALDO/209-382_[41-56] | 4183 | 0.0164% |
| HAFISYSYSDADWVRG | F6PY71_XENTR/645-786_[2-17] | 4157 | 0.0163% |
| YASSSWAARTLASYFN | A0A2P5BHT4_TREOI/23-165_[66-81] | 4136 | 0.0162% |
| GKHSPEGTSSVEYFVH | L8Y5P5_TUPCH/292-467_[8-23] | 3991 | 0.0157% |
| EFPSILRFITIADYTN | A0A2Y9LJK0_DELLE/163-295_[100-115] | 3940 | 0.0155% |
| STVLNESLKNRGINTF | A0A2G3AS97_CAPCH/90-165_[3-18] | 3878 | 0.0152% |
| LLSITKYPIGLESRVQ | G7L9E6_MEDTR/10-205_[168-183] | 3870 | 0.0152% |
| RLALKEVGNISGWHFH | A0A2I4ERM2_JUGRE/26-210_[131-146] | 3857 | 0.0151% |
| REDIEKVQGWRDALTK | A0A498K2Y2_MALDO/23-202_[120-135] | 3839 | 0.0151% |

Table S5. The redundant peptides in the T-Surf P3 library.

| **T-Surf P3** (Total NGS count: 11,882,223) | | | |
| --- | --- | --- | --- |
| **Peptide sequence** | **Peptide ID** | **NGS count** | **Population** |
| WVMSELIPQVEGEQGW | W5LU97_ASTMX/805-959_[14-29] | 4615 | 0.0388% |
| EDEAQEHYAAQDHQTE | H2ME49_ORYLA/793-971_[155-170] | 2921 | 0.0246% |
| NMAGWHVPVTRSKSKA | W9S3W0_9ROSA/4-183_[137-152] | 2304 | 0.0194% |
| VKRLDHGDSIPEELVK | A0A3Q7HJP3_SOLLC/14-198_A0A3Q7HJP3.1_[4] | 2295 | 0.0193% |
| SYANEDRGWVLDHLLP | A0A2A4K1U2_HELVI/595-746_[6-21] | 2138 | 0.0180% |
| YYRVRKLVKKLTYLSW | A0A3Q3GVF1_9LABR/763-917_[103-118] | 2131 | 0.0179% |
| WAFSNGHEAKFIHNIV | A0A251MW86_PRUPE/10-188_[142-157] | 2111 | 0.0178% |
| RELSKILEAMEARGGV | A0A2P6PKL6_ROSCH/19-206_A0A2P6PKL6.1_[7] | 2025 | 0.0170% |
| SENYAFSTWALDELVK | A0A445BMJ9_ARAHY/58-235_A0A445BMJ9.1_[6] | 1893 | 0.0159% |
| WREALAEVAALAGMVL | A0A251QIA6_PRUPE/17-198_[129-144] | 1844 | 0.0155% |
| EELVKIMERRRSFGQV | A0A2P5CSC9_PARAD/21-195_A0A2P5CSC9.1_[7] | 1827 | 0.0154% |
| NTDQSPPYNSRFWKSL | A0A3Q0DKW3_TARSY/78-241_[124-139] | 1812 | 0.0152% |
| FYGRDTRRGFTDHLRA | A0A2I4E9C4_JUGRE/24-205_[7-22] | 1806 | 0.0152% |

Table S6. Characteristics of human TLRs, related modulators, and inhibitory peptides.

| **TLR** | **Uniprot ID** | **TLR active form** | **Signaling adaptors** | **PDB ID** | **Related modulators** **(known inhibitors)** | **TDIPs identified in this study^a^** | **TIR-derived inhibitory peptides reported elsewhere**^1^ |
| --- | --- | --- | --- | --- | --- | --- | --- |
| TLR1 | Q15399 | TLR1-TLR2, TLR1-TLR10 | MyD88 | 2Z7X (Ectodomain of TLR1-TLR2 heterodimer), 1FYV (TIR) | CU-CPT22^2^ | TDIP1~8, TDIP10, TDIP16~17, TDIP20 | MIP2^3^ |
| TLR2 | O60603 | TLR1-TLR2, TLR2-TLR2, TLR2-TLR6, TLR2-TLR10 | MyD88, TIRAP | 2Z7X (Ectodomain of TLR1-TLR2 heterodimer), 1FYW (TIR) | C29^4^, CU-CPT22^2^, Lipolanthionine peptides^5^, LL-37^6^, OxPAPC^7^ | TDIP1~8, TDIP10, TDIP16~17, TDIP20 | 1R10, 2BB, 2R1, 2R3, 2R9, 4BB, 4R9, 6R10, 6R9, IR9, MIP2^3^, TR3, TR6 |
| TLR3 | O15455 | TLR3-TLR3 | TRIF | 1ZIW (Ectodomain) | Not reported | Not detected | MIP2^3^ |
| TLR4 | O00206 | TLR4/MD2-TLR4/MD2, TLR4-TLR6 | MyD88, TRIF, TRAM, TIRAP | 3FXI (Ectodomain) | Benzylammonium lipid^8^, CRX-526^9^, E5564^10^, LL37^6^, OxPAPC^7^, TAK-242^11^ | TDIP1, TDIP3~6, TDIP8, TDIP10, TDIP15~17, TDIP20 | 2BB, 2R9, 4BB, 4R9, 4αE, MIP2^3^, TAP2^12^, TF5, TM4-ΔC, TM6, TR11, TR3, TR5, TR9 |
| TLR5 | O60602 | TLR5-TLR5 | MyD88 | 3J0A (Full-length) | Not reported | TDIP14 | 5R667^13^ |
| TLR6 | Q9Y2C9 | TLR2-TLR6, TLR4-TLR6 | MyD88, TIRAP | 4OM7 (TIR) | C29^14^ | Not tested | MIP2^3^ |
| TLR7 | Q9NYK1 | TLR7-TLR7 | MyD88 | 7CYN (Full-length) | IRS954^15,16^ | TDIP2~8, TDIP10, TDIP12~13, TDIP15~16, TDIP19~21 | 2R9, 7R9^17^, 7R11^17^, MIP2^3^ |
| TLR8 | Q9NR97 | TLR8-TLR8 | MyD88 | 3W3G (Ectodomain) | CU-CPT9a^18^ | Not detected | - |
| TLR9 | Q9NR96 | TLR9-TLR9 | MyD88 | - | IRS954^15,16^ | TDIP9 | 2R9, 9R11, 9R3, 9R34-ΔN, 9R9, MIP2^3^ |
| TLR10 | Q9BXR5 | TLR1-TLR10 TLR2-TLR10 TLR10-TLR10 | MyD88 | 2J67 (TIR) | Not reported | Not tested | - |

a) TDIPs showing greater than 50% inhibition effect compared to each TLR activator condition (treated with PBS) were selected from Figure. 2c.

Data S1.

Final T-Surf library (related to Figure. 1).

Data S2.

NGS data of 4R selection against MAL and MyD88 (related to Figure. 1).

Data S3.

Top 11 peptides from each selected pool (related to Figure. 1).

Data S4.

Peptide ranking after grouping (related to Figure. 1).

**References**

1. Toshchakov, V. Y. & Javmen, A. Targeting the TLR signalosome with TIR domain-derived cell-permeable decoy peptides: The current state and perspectives. *Innate Immun.* **2020**, 26, 35–47.

2. Cheng, K., Wang, X., Zhang, S. & Yin, H. Discovery of small-molecule inhibitors of the TLR1/TLR2 complex. *Angew. Chem. Int. Ed. Engl.* **2012,** 51, 12246–12249.

3. Shah, M. *et al*. The αC helix of TIRAP holds therapeutic potential in TLR-mediated autoimmune diseases. *Biomaterials* **2020**, 245, 119974.

4. Mistry, P. *et al*. Inhibition of TLR2 signaling by small molecule inhibitors targeting a pocket within the TLR2 TIR domain. *Proc. Natl. Acad. Sci. U. S. A.* **2015**, 112, 5455–5460.

5. Seyberth, T., Voss, S., Brock, R., Wiesmüller, K. H. & Jung, G. Lipolanthionine peptides act as inhibitors of TLR2-mediated IL-8 secretion. Synthesis and structure-activity relationships. *J. Med. Chem.* **2006**, 49, 1754–1765.

6. Di Nardo, A. *et al.* Cathelicidin antimicrobial peptides block dendritic cell TLR4 activation and allergic contact sensitization. *J. Immunol.* **2007**, 178, 1829–1834.

7. Erridge, C., Kennedy, S., Spickett, C. M. & Webb, D. J. Oxidized phospholipid inhibition of toll-like receptor (TLR) signaling is restricted to TLR2 and TLR4: Roles for CD14, LPS-binding protein, and MD2 as targets for specificity of inhibition. *J. Biol. Chem.* **2008**, 283, 24748–24759.

8. Piazza, M. *et al*. Evidence of a specific interaction between new synthetic antisepsis agents and CD14. *Biochemistry* **2009**, 48, 12337–12344.

9. Bazin, H. G. *et al*. The ‘Ethereal’ nature of TLR4 agonism and antagonism in the AGP class of lipid A mimetics. *Bioorg. Med. Chem. Lett.* **2008**, 18, 5350–5354.

10. Kim, H. M. *et al.* Crystal structure of the TLR4-MD-2 complex with bound endotoxin antagonist eritoran. *Cell* **2007**, 130, 906–917.

11. Ii, M. *et al*. A novel cyclohexene derivative, ethyl (6R)-6-[N-(2-chloro-4-fluorophenyl) sulfamoyl]cyclohex-1-ene-1-carboxylate (TAK-242), selectively inhibits toll-like receptor 4-mediated cytokine production through suppression of intracellular signaling. *Mol. Pharmacol.* **2006**, 69, 1288–1295.

12. Park, S. *et al*. TLR4/MD2 specific peptides stalled in vivo LPS-induced immune exacerbation. *Biomaterials* **2017**, 126, 49–60.

13. Javmen, A. *et al.* TLR5-derived, TIR-interacting decoy peptides to inhibit TLR signaling. *J. Immunol.* **2023**, 210, 1419–1427.

14. Takeuchi, O. *et al*. Discrimination of bacterial lipoproteins by toll-like receptor 6. *Int. Immunol.* **2001**, 13, 933–940.

15. Wang, D. *et al*. Oligodeoxyribonucleotide-based antagonists for toll-like receptors 7 and 9. *J. Med. Chem.* **2009**, 52, 551–558.

16. Barrat, F. J. et al. Treatment of lupus-prone mice with a dual inhibitor of TLR7 and TLR9 leads to reduction of autoantibody production and amelioration of disease symptoms. Eur. J. Immunol. **2007**, 37, 3582-3586.
